# Real-Time Artificial Intelligence Versus Standard Colonoscopy in the Early Detection of Colorectal Cancer: A Systematic Review and Meta-Analysis

**DOI:** 10.3390/healthcare13192517

**Published:** 2025-10-03

**Authors:** Abdullah Sultany, Rahul Chikatimalla, Adishwar Rao, Mohamed A. Omar, Abdulkader Shaar, Hassam Ali, Fariha Hasan, Sheza Malik, Saqr Alsakarneh, Dushyant Singh Dahiya

**Affiliations:** 1 Department of Internal Medicine, Guthrie Robert Packer Hospital, Sayre, PA 18840, USA; abdullah.sultany@guthrie.org; 2 Miller School of Medicine, University of Miami, Miami, FL 33125, USA; chrahul27@gmail.com (R.C.);; 3 Department of Gastroenterology, University of Kansas, Wichita, KS 67214, USA; 4 ECU Health Medical Center, Brody School of Medicine, Greenville, Department of Gastroenterology, Hepatology & Nutrition, Greenville, NC 27834, USA; 5 Department of Internal Medicine, Cooper University Hospital, Camden, NJ 08103, USA; 6 Division of Digestive Diseases, Emory University School of Medicine, Atlanta, GA 30322, USA; 7 Department of Internal Medicine, University of Missouri-Kansas City, Kansas City, MO 64108, USA; 8 Division of Gastroenterology, Hepatology & Motility, The University of Kansas School of Medicine, Kansas City, KS 66160, USA

**Keywords:** artificial intelligence, colonoscopy, computer-aided detection, polyp detection rate, adenoma detection rate

## Abstract

**Background:** Colonoscopy remains the gold standard for colorectal cancer screening. Deep learning systems with real-time computer-aided polyp detection (CADe) demonstrate high accuracy in controlled research settings and preliminary randomized controlled trials (RCTs) report favorable outcomes in clinical settings. This study aims to evaluate the efficacy of AI-assisted colonoscopy compared to standard colonoscopy focusing on Polyp Detection Rate (PDR) and Adenoma Detection Rate (ADR), and to explore their implications for clinical practice. **Methods:** A systematic search was conducted using multiple indexing databases for RCTs comparing AI-assisted to standard colonoscopy. Random-effect models were utilized to calculate pooled odds ratios (ORs) with 95% confidence intervals. The risk of bias was assessed using the Cochrane Risk of Bias Tool, and heterogeneity was quantified using I statistics. **Results:** From 22,762 studies, 12 RCTs (n = 11,267) met the inclusion criteria. AI-assisted colonoscopy significantly improved PDR (OR 1.31, 95% CI 1.08–1.59, *p* = 0.005), despite heterogeneity among studies (I^2^ = 79%). While ADR showed improvement with AI-assisted colonoscopy (OR 1.24, 95% CI, 0.98–1.58, *p* = 0.08), the result was not statistically significant and had high heterogeneity (I^2^ = 81%). **Conclusions:** AI-assisted colonoscopy significantly enhances PDR, highlighting its potential role in colorectal cancer screening programs. However, while an improvement in the ADR was observed, the results were not statistically significant and showed considerable variability. These findings highlight the promise of AI in improving diagnostic accuracy but also point to the need for further research to better understand its impact on meaningful clinical outcomes.

## 1. Introduction

Colorectal cancer (CRC) remains a leading cause of cancer-related mortality worldwide but is substantially reducible through timely and effective screening. Colonoscopy plays a crucial role by enabling the detection and removal of adenomatous polyps, with studies showing a 57% reduction in advanced-stage interval CRC and a 62% reduction in fatal interval CRC among patients of physicians with high adenoma detection rates [1]. However, the preventive success of colonoscopy relies heavily on the quality of the examination, particularly the thoroughness of polyp detection. The adenoma detection rate (ADR), defined as the proportion of individuals undergoing colonoscopy in whom at least one adenoma is identified, serves as the foremost quality indicator of colonoscopy performance [2]. Notably, higher ADRs, which reflect greater thoroughness in polyp detection during colonoscopy, are strongly linked to reduced risks of interval CRC. For every 1% increase in an endoscopist’s ADR, a patient’s risk of developing fatal interval CRC after colonoscopy decreases by approximately 5% [1]. To ensure adequate examination standards, current clinical guidelines recommend benchmark ADR targets of 35% or higher in average-risk screening populations [3].

Despite adherence to established quality standards, colonoscopies do not detect every precancerous lesion. A substantial fraction of polyps including adenomas may be missed during a single examination due to factors such as suboptimal viewing or subtle lesion morphology [4]. Meta-analyses of tandem colonoscopy studies estimate that approximately one in four adenomas is missed during an index colonoscopy [4,5]. These overlooked lesions are believed to account for a significant proportion of post-colonoscopy CRC cases, known as interval cancers [6,7]. Moreover, variability in detection performance among endoscopists further highlights the need for adjunctive methods to enhance polyp and adenoma detection rates.

Artificial intelligence (AI) has emerged as a promising tool to enhance endoscopic detection in recent years [8]. AI-based real-time computer-aided detection (CADe) systems use advanced computer vision and machine learning algorithms to analyze the endoscopic video feed during the procedure. As the endoscopist navigates the colon, the CADe system continuously processes each video frame in real time, automatically identifying and highlighting suspicious areas that may represent polyps or other mucosal abnormalities. These alerts act as a second observer and draw the endoscopist’s attention to lesions that might otherwise be overlooked, especially small or subtle polyps, and allow the endoscopist to assess and decide whether a lesion requires further inspection or removal [9]. Early clinical studies of CADe in colonoscopy have shown encouraging results [10]. For example, a large multicenter randomized trial in the United States reported a 27% relative increase in adenomas detected per colonoscopy with an AI device; approximately one additional adenoma was found for every 4–5 patients screened [5]. Similarly, other trials have observed significant improvements in polyp detection with AI with a reported doubling of ADR with the use of AI [11].

Although individual RCTs have demonstrated the potential of real-time AI-assisted colonoscopy to enhance polyp and adenoma detection, findings across studies remain heterogeneous, and a clear consensus on its clinical utility is lacking. Furthermore, prior reviews have often included non-randomized designs or earlier-generation systems with limited generalizability.

To address this gap, we conducted a systematic review and meta-analysis of recent randomized controlled trials focused specifically on real-time CADe systems during colonoscopy. Our study provides the most up-to-date and comprehensive pooled estimates of the impact of AI assistance on key quality indicators, polyp detection rate (PDR) and adenoma detection rate (ADR). Our aim was to quantitatively assess whether CADe improves polyp detection rate (PDR) and ADR, and to evaluate the clinical significance of integrating AI into routine colonoscopic practice for CRC prevention.

## 2. Methods

### 2.1. Study Design

**Figure 1:**
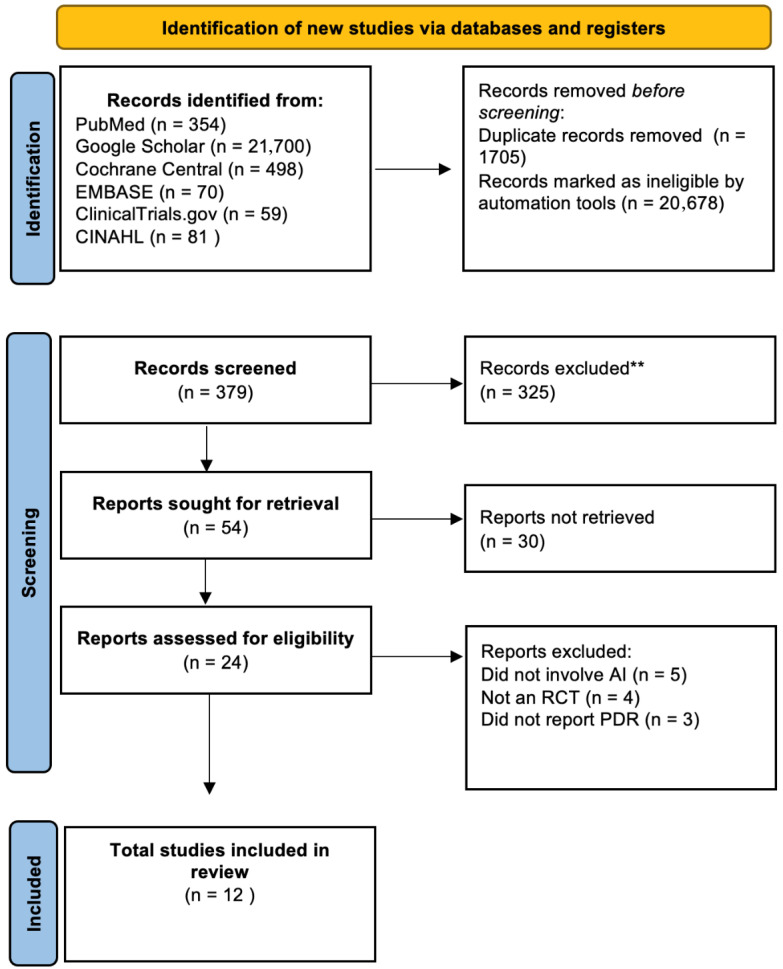
PRISMA flow diagram representing study selection. **—Studies excluded due to lack of comparative data, no relevant outcomes, inappropriate design (non-RCT/observational), editorials, or duplicate publications.

This meta-analysis was conducted in accordance with the guidelines specified in the Preferred Reporting Items for Systematic Reviews and Meta-Analyses (PRISMA) Statement [12]. As this study constitutes a meta-analysis, ethical approval and informed consent were not required. The process of selecting studies is represented in the PRISMA flow diagram (Figure 1 and Supplemental S1). The protocol for this systematic review and meta-analysis was registered in the PROSPERO database (CRD420251112020).

### 2.2. Identification of Studies

A comprehensive search of randomized controlled trials (RCTs) was conducted using several databases including PubMed, Embase, Cochrane Central Register of Controlled Trials (CENTRAL), ClinicalTrials.gov, and Google Scholar. The search period extended from January 2019 to November 2024 and included all published English language RCTs that met the inclusion criteria using specific keywords “Artificial Intelligence”, “Computer-Aided Detection (CADe)”, “Machine Learning”, “Deep Learning”, “Colonoscopy”, “Polyp Detection Rate”, “Adenoma Detection Rate”, and “Colorectal Cancer”. Manual citation searching of references from retrieved articles was also performed to identify any relevant studies that were missed through database search. The search strategy, research question, PICO, MeSH, and keywords are described in Supplemental S2. In all included studies, AI-assisted colonoscopy refers to procedures performed by medical doctors using real-time computer-aided detection (CADe) systems. These systems provide visual alerts (e.g., bounding boxes) on the endoscopic display to assist the endoscopist in identifying potential lesions. Final interpretation and management decisions were always made by the endoscopist.

### 2.3. Inclusion and Exclusion Criteria

Only RCTs comparing artificial intelligence-assisted colonoscopy (AI-assisted, specifically computer-aided detection systems) with standard colonoscopy were included. Studies were included if they (1) involved adult patients (>18 years) undergoing colonoscopy for screening or diagnostic purposes. (2) Reported at least one of the outcomes of interest: PDR or ADR. (3) Randomized patients to either an AI-assisted colonoscopy or a control group without AI assistance. Studies were excluded if they (1) were quasi-randomized or non-randomized. (2) Focused solely on the detection of serrated lesions or used retrospective designs. (3) Did not report PDR or ADR as outcomes. (4) Lacked adequate data for extraction or analysis. Endoscopist experience was reported by the original study authors. In all included trials, procedures were performed by physicians described as experienced, typically defined as having performed >1000 lifetime colonoscopies, being board-certified, or meeting national competency thresholds.

### 2.4. Data Extraction and Quality Assessment

Two independent reviewers (RC, AS) screened titles and abstracts for eligibility, followed by full-text assessments. Disagreements were resolved through discussion and by an independent third reviewer (AR). Data on study design, patient demographics, intervention and control procedures, and primary outcomes (PDR and ADR) were extracted.

**Figure 2:**
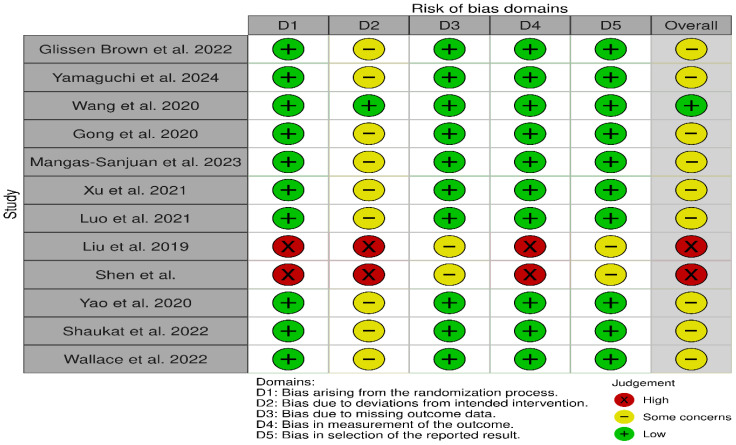
Risk of bias (ROB) assessment for randomized control trials [5,7,9,11,14,15,16,17,18,19,20,21].

The methodological quality and risk of bias of the included studies were assessed using the Cochrane Risk of Bias Tool (RoB 2.0) (https://methods.cochrane.org/bias/resources/rob-2-revised-cochrane-risk-bias-tool-randomized-trials, accessed on 14 September 2025) [13]. Domains evaluated included random sequence generation, allocation concealment, blinding, incomplete outcome data, and selective reporting. Figure 2 shows the risk of bias table.

### 2.5. Outcomes

A polyp refers to any mucosal protrusion identified during colonoscopy and can be neoplastic (e.g., adenomas, carcinomas) or non-neoplastic (e.g., hyperplastic or inflammatory polyps). An adenoma is a histologically confirmed neoplastic polyp and a precursor lesion in the adenoma-carcinoma sequence of colorectal cancer. Adenomas with in situ adenocarcinoma or high-grade dysplasia are classified as advanced neoplasia. The primary outcomes for this study were (1) ADR, defined as the proportion of individuals who underwent a complete colonoscopy and in whom at least one adenoma was identified and excised. (2) PDR: the proportion of individuals who undergo a complete colonoscopy and have at least one polyp identified. The secondary outcome of interest was withdrawal time, meaning the duration spent examining the colonic mucosa as the endoscope is pulled back during a colonoscopy. While Adenoma Miss Rate (AMR) was reported in some included trials, it was not used as an inclusion criterion for study selection.

### 2.6. Data Synthesis and Analysis

In individual trials, we estimated risk ratios (RRs) for dichotomous outcomes and mean differences for continuous outcomes, together with their 95% confidence intervals (CIs). We evaluated the variability in intervention effects across primary studies by employing the x^2^ test (Cochran Q) and the I^2^ statistic. Pooled estimates were calculated using the DerSimonian and Laird random-effects model [22]. We interpreted I^2^ values of 25%, 50%, and 75% as representing low, moderate, and high heterogeneity, respectively [23].

Sensitivity analyses were conducted using the leave-one-out method for the main outcomes, specifically ADR and PDR, to assess the impact of each individual study on the overall effect estimate and evaluate the robustness of pooled estimates. Additionally, we carried out a sensitivity analysis for withdrawal time by omitting studies that examined systems controlling the endoscope withdrawal time. We explored publication bias using funnel plots. We rated the quality of evidence according to the Grades of Recommendation, Assessment, Development and Evaluation approach (GRADE) [24]. Supplemental S3 shows the GRADE assessment of included studies

Publication bias was assessed visually through funnel plots and quantitatively using Egger’s test and the Trim-and-Fill method for asymmetry. We used Stata version 16 (StataCorp LP, StataCorp, College Station, TX, USA and RevMan 5.3 (The Cochrane Collaboration, Copenhagen, Denmark) for performing statistical analysis for this study.

## 3. Results

### 3.1. Study Selection and Characteristics

A systematic search from multiple sources produced a total of 22,762 records. After duplicate studies were removed and titles and abstracts were screened, 57 articles were assessed for inclusion in the study. Twelve RCTs with a total of 11,267 patients met the inclusion criteria and were subsequently included in the meta-analysis [5,7,9,11,14,15,16,17,18,19,20,21].

The trials included in this review took place from 2019 to 2024 in several countries, although the majority (seven) were performed in China. The studies all matched standard colonoscopy to real-time artificial intelligence (AI)–assisted colonoscopy for colorectal adenoma or polyp detection. Major study features, such as population, clinical setting, AI systems, primary outcome, and endoscopist level, are reported in Table 1.

Participant age varied from 49 to 64.5 years, with an overall balanced gender split in intervention groups. Most trials excluded a history of prior colorectal surgery, hereditary cancer syndromes, or inflammatory bowel disease to achieve homogeneity in samples. Endoscopy was carried out in all cases by experienced endoscopists, conventionally regarded as having performed more than 1000 lifetime colonoscopies or as board-certified. Because real-time AI overlays in the procedure can be seen, blinding of the endoscopist was not possible in any of these included trials. Technical specifications, which go beyond those in trials, about systems of AI and deploy methods can be seen in Supplemental S4.

### 3.2. Polyp Detection Rate

**Figure 3:**
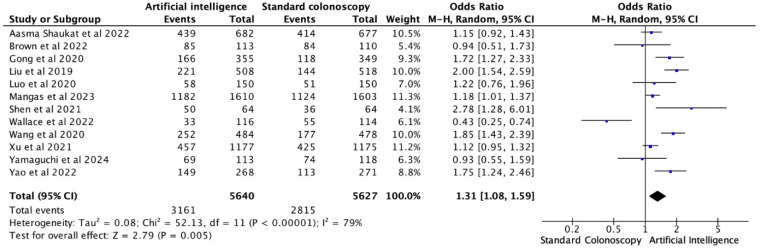
Forest plot for PDR comparing AI-assisted versus standard colonoscopy [5,7,9,11,14,15,16,17,18,19,20,21].

All 12 RCTs provided PDR as the proportion of procedures in which a polyp was found. The pooled analysis showed a significantly higher PDR for AI-assisted compared with standard colonoscopy (OR = 1.31; 95% CI, 1.08–1.59; *p* = 0.005), representing a 31% higher chance of polyp detection in cases where AI support was used. Considerable heterogeneity was noted among the studies (I^2^ = 79%, τ^2^ = 0.08, *p* < 0.0001). The overall effect was confirmed by performing leave-one-out sensitivity analysis, which demonstrated a consistent statistical significance (Figure 3 and Supplemental S5).

To assess the potential for publication bias, Egger’s regression test was conducted (*p* = 0.883). Additionally, a visual review of the funnel plot showed a symmetrical distribution of effect sizes, suggesting a low likelihood of small-study effects. Trim-and-Fill likewise imputed no missing studies, with the adjusted effect size (logOR = 0.265, 95% CI: 0.034 to 0.497) unchanged from the observed, confirming the absence of publication bias. (Supplementals S6–S8).

### 3.3. Adenoma Detection Rate

**Figure 4:**
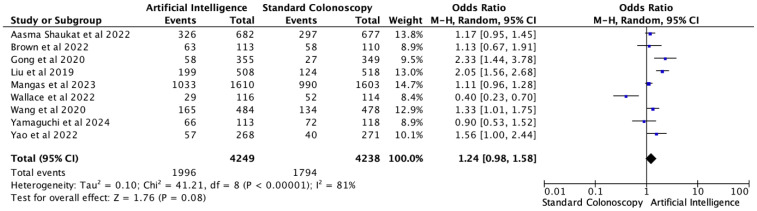
Forest plot for ADR comparing AI-assisted versus standard colonoscopy [5,7,9,11,14,15,16,18,21].

ADR was described in nine of the 12 trials. The pooled analysis (Figure 4) revealed a numerical but not statistically significant benefit in favor of AI-assisted colonoscopy (OR = 1.24; 95% CI, 0.98–1.58; *p* = 0.08). Overall, 1996 adenomas were identified in the AI group and 1794 in the control arm.

There was substantial between-study heterogeneity (I^2^ = 81%, τ^2^ = 0.10, *p* < 0.0001). Stability was tested by sensitivity analysis using the leave-one-out approach; direction and magnitude of effect remained consistent in all iterations. Evaluation of small-study effects by Egger’s regression test (*p* = 0.956) did not suggest evidence of publication bias. In addition, this was also supported by the funnel plot’s symmetrical distribution implying the reliability of the pooled estimate despite heterogeneity. Trim-and-Fill analysis identified no imputed studies, and the adjusted pooled effect size (logOR = 0.205, 95% CI: −0.098 to 0.508) was identical to the observed estimate, indicating no evidence of publication bias for ADR (Supplementals S9–S12).

### 3.4. Withdrawal Time

**Figure 5:**
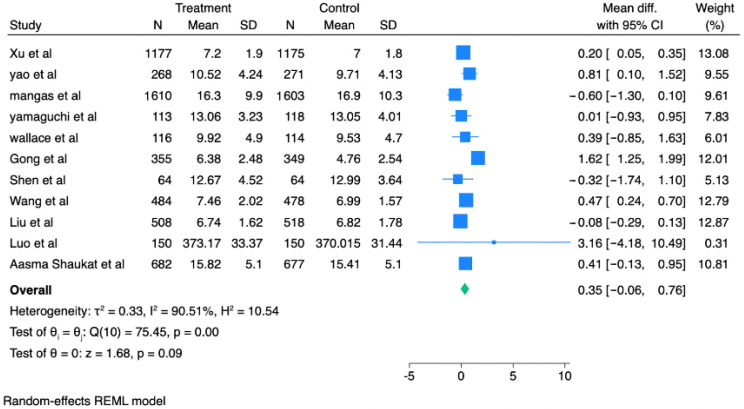
Forest plot comparing mean withdrawal time between AI-assisted and standard colonoscopy [5,7,9,11,15,16,17,18,19,20,21].

Withdrawal time was available in 11 of the RCTs included. Analysis of the pooled estimate revealed a modest and non-significant prolonged mean withdrawal time in AI-assisted colonoscopy versus standard colonoscopy (Figure 5). There was a mean difference of 0.35 min (95% CI, −0.06 to 0.76; *p* = 0.09; I^2^ = 90.5%).

A leave-one-out sensitivity analysis was used to test the stability of this result (Supplemental S13). This test showed that the averaged mean difference kept the same comparable boundaries (0.19 to 0.46 min) and remained non-significant for any combination except when the study of Mangas-Sanjuan et al. was removed (*p* = 0.026). Visual inspection of the funnel plot showed no evidence of potential publication bias [18].

### 3.5. Subgroup Analyses

To investigate sources of heterogeneity, we conducted a priori subgroup analyses stratified by AI platform type (commercial vs. custom/in-house). Commercial systems were defined as platforms marketed or holding regulatory approval (e.g., GI Genius, DISCOVERY, later commercialized versions of EndoAngel), reflecting tools validated for broad clinical deployment. Custom/in-house systems were investigational, locally developed models evaluated primarily in single-center or limited multicenter research settings without broad regulatory clearance. Based on these criteria, five trials [5,7,14,18,21] were classified as commercial, and seven [9,11,15,16,17,19,20] as custom/in-house. Random-effects models were applied within each subgroup, and between-group differences were tested using Q statistics. This stratification was performed for both ADR and PDR outcomes to assess whether platform maturity contributed to variability in effect estimates.

**Figure 6:**
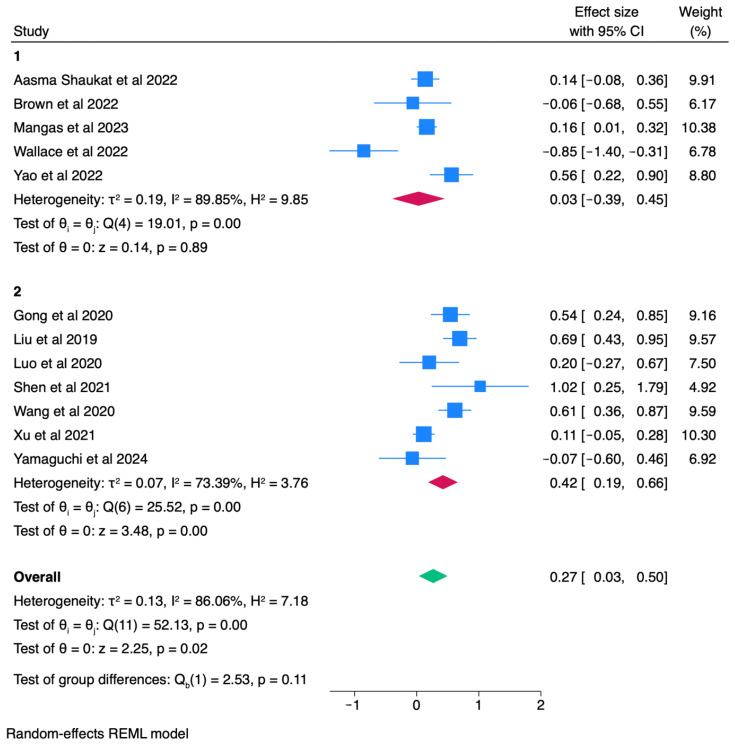
Subgroup analysis of Polyp Detection Rate (PDR) by AI platform type [5,7,9,11,14,15,16,17,18,19,20,21].

**Figure 7:**
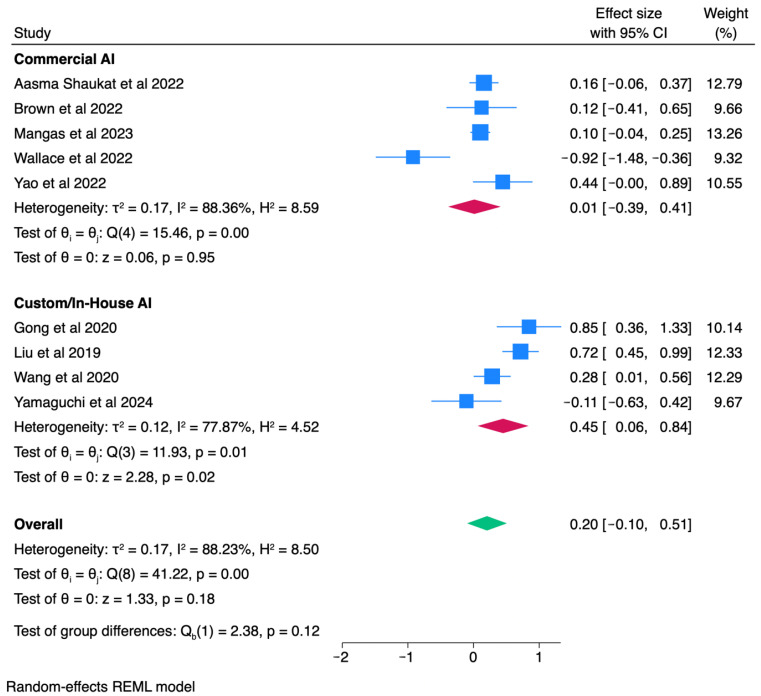
Subgroup analysis of ADR by AI platform type [5,7,9,11,14,15,16,18,21].

Subgroup analysis by platform type demonstrated notable differences in effect estimates. For PDR, custom/in-house CADe systems (7 trials) were associated with a significant improvement (logOR = 0.42, 95% CI: 0.19–0.66; *p* < 0.01; I^2^ = 73.4%), whereas commercial systems (5 trials) showed no significant benefit (logOR = 0.03, 95% CI: –0.39–0.45; I^2^ = 89.9%). The between-group comparison did not reach statistical significance (Qb = 2.53, *p* = 0.11) (Figure 6). A similar pattern was observed for ADR, where custom platforms yielded significant gains (logOR = 0.45, 95% CI: 0.06–0.84; *p* = 0.02; I^2^ = 77.9%), while commercial systems demonstrated no effect (logOR = 0.01, 95% CI: −0.39–0.41; I^2^ = 88.4%). Collectively, these findings indicate that system maturity may contribute to heterogeneity, with custom/in-house CADe platforms exhibiting stronger performance than commercial counterparts, although between-group differences were not statistically significant (Figure 7).

## 4. Discussion

In this meta-analysis, the use of AI yielded a 31% relative increase in PDR as well as a 24% rise in ADR though this finding was statistically non-significant. This result shows that the use of CADe in colonoscopies resulted in fewer missed lesions but that the rate of detecting histologically confirmed polyps, while higher, did not significantly differ from when not used. The increased PDR relative to ADR is likely because CADe primarily picks up diminutive tubular adenomas or serrated polyps that the endoscopist might have otherwise missed. The substantial heterogeneity (81%) noted in the pooled ADR could be attributed to variation at the trial level, patients, surgery modalities, and AI models.

In a 2021 meta-analysis by Hassan et al., the use of CADe showed significantly improved ADR by 44% though this was performed in 5 studies only that were entirely Chinese RCTs [25]. In our study, the introduction of four large Western-based, multicenter RCTs led to a shrunken pooled ADR effect that was directionally positive but statistically non-significant [5,7,14,18]. This finding suggests that previous estimates were possibly inflated by small, single-center studies. Notably though, none of the trials reported a decrease in the adenoma detection with AI and all either met or trended toward improved polyp findings.

Trials in which baseline ADR was moderate (20–30%) saw the most significant relative gains with AI (often ~50–100% relative increase) [11,16]. In contrast, trials conducted in high-performing settings (ADR ≥ 45–60%) showed more modest improvements or no significant ADR change [5]. The lack of improvement in the yield of advanced adenoma in a high-ADR setting warrants consideration [18]. It suggests that once a certain expert-level ADR is achieved, simply adding a detection device may mainly reflect diminishing returns (tiny polyps of uncertain clinical significance). This underscores the importance of tailoring expectations of AI as its impact is likely greatest for endoscopists or environments where some adenomas are currently being missed. In lower ADR contexts or among trainees, as shown by Yamaguchi et al., CADe serves as a valuable safety net that raises the baseline detection closer to an expert level [14,15].

Another emerging insight is the importance of human technique alongside AI. The factorial study by Yao et al. demonstrated that enforcing a proper withdrawal technique (adequate time and mucosal exposure) can independently boost ADR to a level comparable with AI’s effect [21]. This finding underscores that AI is most effective as an adjunct to, not a replacement for, high-quality colonoscopy. Endoscopists should continue to practice optimal intubation, withdrawal, and visualization techniques. When CADe is combined with such quality measures, the highest detection rates are achieved as seen in Yao et al., where CADe + quality yielded ~31% ADR vs. ~15–24% in standard practice [21]. Thus, AI can be viewed as a complementary tool that augments the endoscopist’s vigilance and mitigates perceptual errors such as fatigue or inattention-related misses.

Our results found that the withdrawal time did not significantly vary between CADe and controls. However, the high level of heterogeneity (90.5%) is probably due to differences in procedures, AI-alert systems, and reporting tools. It should be noted that the article by Luo et al. had outliers for withdrawal time, which skewed heterogeneity [17]. As such, taken together, these results indicate that AI-assisted colonoscopy results in only slightly longer withdrawal time, probably because of increased vigilance in mucosal inspection, which does not possess statistical significance and does not seem to affect procedural efficiency in a negative manner.

High heterogeneity across trials has been a key limitation in prior reviews. Our subgroup analysis indicates that platform type may contribute; custom/in-house CADe systems consistently showed greater improvements in detection rates compared to commercial platforms. Although the between-group difference test was not statistically significant, the trend highlights that technical design and training datasets could influence outcomes. This stratification helps contextualize heterogeneity and provides direction for future evaluations of AI-assisted colonoscopy.

This study has several limitations that merit discussion. First, the presence of inter-study heterogeneity must be acknowledged. The RCTs differed in patient populations (some focusing on screening exams, others including high-risk or surveillance cohorts), endoscopist expertise and baseline ADR, and the specific CADe platforms used. These differences likely contributed to variability in effect sizes. Second, most trials (and all in our analysis) were open label with respect to the endoscopist, which could introduce performance bias. Third, our meta-analysis endpoints (PDR and ADR) are intermediate outcomes; while they are validated surrogates, we cannot directly conclude that AI-assisted colonoscopy will reduce interval cancer incidence or improve patient survival. The hope is that by catching more adenomas now, fewer cancers will develop later—a premise supported by our current understanding of ADR-outcomes correlation. A key limitation of our analysis is that ADR and PDR serve as surrogate quality indicators and do not directly capture long-term outcomes such as colorectal cancer incidence, mortality, or cost-effectiveness, which remain important areas for future investigation. Although all included trials were randomized, ensuring general balance across groups, residual baseline differences cannot be fully excluded and propensity score matching was not applicable in this context. Nevertheless, long-term follow-up data are needed. Finally, the applicability of these findings to all practice settings should be considered. As included RCTs did not employ tandem colonoscopy designs, inter-patient variability cannot be entirely excluded. However, randomization across trials minimizes this risk by balancing baseline characteristics between groups. Many of the trials were conducted in academic or high-volume centers with expert endoscopists, and the absolute benefit of AI might be even greater in community practice where ADRs can be lower.

Moving forward, ongoing research will clarify how much AI-driven increases in adenoma detection ultimately impact patient survival and how best to implement CADe across different practice settings. Future iterations of AI may better recognize flat lesions or provide real-time histologic characterization (integrating CADe for polyp pathology prediction), which could further enhance decision-making by helping endoscopists prioritize which polyps to remove. These advances and evidence from longer-term outcome studies will clarify the ultimate value of AI assistance in colonoscopy. Preliminary data from this study were presented as a conference abstract at Digestive Disease Week (DDW) 2025 [26].

## 5. Conclusions

In summary, real-time computer-assisted colonoscopy (with CADe systems) significantly enhances the detection of colorectal polyps. It demonstrates a potential yet modest enhancement in polyp detection versus standard colonoscopy. By highlighting subtle lesions that may otherwise go undetected, AI has the potential to add quality to the performance of colonoscopic examinations.

## Figures and Tables

**Table 1:** Baseline characteristics of the included randomized controlled trial.

Study	Sample Size (AI/Control)	Males in AI Group, n (%)	Males in Control Group, n (%)	Mean Age in Years (AI/Control)	Primary Outcome
Brown et al., 2022 [14]	116/116	60 (52%)	44 (38%)	61.18/60.51	AMR
Gong et al., 2020 [11]	355/349	187 (52.7%)	158 (45.3%)	56.7/56.7	ADR
Liu et al., 2019 [16]	508/518	264 (51.9%)	287 (55.4%)	N/A	ADR
Luo et al., 2021 [17]	150/150	76 (51%)	76 (51%)	41.3/41.3	ADR
Mangas et al., 2023 [18]	1610/1603	865 (53.7%)	852 (53.2%)	60.7/60.6	AN
Shaukat et al., 2022 [5]	682/677	368 (54.0%)	355 (52.4%)	60.6/59.9	APC
Shen et al., 2021 [19]	64/64	31 (48.4%)	31 (48.4%)	N/A	PDR
Wallace et al., 2022 [7]	116/114	80 (68.97%)	77 (67.54%)	63.0/64.6	AMR
Wang et al., 2020 [9]	484/478	241 (50.0%)	254 (53.0%)	58.1/58.1	ADR
Xu et al., 2021 [20]	1174/1178	603 (51.2%)	595 (50.6%)	50.9/51.7	PDR
Yamaguchi et al., 2024 [15]	113/118	58 (51.3%)	66 (55.9%)	N/A	ADR
Yao et al., 2022 [21]	268/271	121 (45.1%)	114 (42.1%)	50.69/50.85	ADR

Abbreviations: n, number of patients, AI, artificial intelligence, PDR, polyp detection rate, AMR, adenoma miss rate, APC, adenoma per colonoscopy, ADR, adenoma detection rate, AN, advanced neoplasia, N/A, not applicable.

## Data Availability

No new data were created or analyzed in this study.

## Supplementary Materials

The following supporting information can be downloaded at: https://www.mdpi.com/article/10.3390/healthcare13192517/s1, Supplemental S1: Preferred Reporting Items for Systematic Reviews and Meta-analyses (PRISMA) checklist; Supplemental S2: Research Question, PICO, MeSH, Keywords and Search Strategy; Supplemental S3: GRADE assessment of included studies; Supplemental S4: Overview of AI platforms used in the included trials; Supplemental S5: Leave one-out sensitivity testing for PDR; Supplemental S6: Egger’s test for PDR; Supplemental S7: Funnel plots to check for publication bias for PDR; Supplemental S8: Trim-and-Fill Analysis for PDR; Supplemental S9: Leave one-out sensitivity testing for ADR; Supplemental S10: Egger’s test for ADR; Supplemental S11: Funnel plots to check for publication bias for ADR; Supplemental S12: Trim-and-Fill Analysis for ADR; Supplemental S13: Leave one-out sensitivity testing for withdrawal time.

## Abbreviations

ADR	Adenoma Detection Rate
AI	Artificial Intelligence
CADe	Computer-Aided Detection
CI	Confidence Interval
CRC	Colorectal Cancer
OR	Odds Ratio
PDR	Polyp Detection Rate
PRISMA	Preferred Reporting Items for Systematic Reviews and Meta-Analyses
RCT	Randomized Controlled Trial
RoB	Risk of Bias
GRADE	Grades of Recommendation, Assessment, Development and Evaluation
