# Supplementary material for: Real-Time Artificial Intelligence Versus Standard Colonoscopy in the Early Detection of Colorectal Cancer: A Systematic Review and Meta-Analysis
Source: Healthcare (Basel). 2025 Oct 3;13(19):2517. doi: 10.3390/healthcare13192517 (PMC12524564; doi:10.3390/healthcare13192517)

Supplemental File

Title: Real-Time Artificial Intelligence Versus Standard Colonoscopy In The Early Detection of Colorectal Cancer: A Systematic Review and Meta-Analysis

Authors: Abdullah Sultany MD[1,†], Rahul Chikatimalla MD[2,†], Adishwar Rao MD MPH[1], Mohamed A Omar MD[3], Abdulkader Shaar MD[2], Hassam Ali MD[4], Fariha Hasan MD[5], Sheza Malik MD[6], Saqr Alsakarneh MD[7], Dushyant Singh Dahiya MD[8]

Affiliations:
[1]Department of Internal Medicine, Guthrie Robert Packer Hospital, Sayre, PA 18840, USA
[2]University of Miami, Miami, FL 33125, USA
[3]Department of Gastroenterology, University of Kansas - Wichita, Wichita, KS 67214, USA
[4]Department of Gastroenterology, Hepatology & Nutrition, ECU Health Medical Center/Brody School of Medicine, Greenville, NC 27834, USA
[5]Department of Internal Medicine, Cooper University Hospital, Camden, NJ 08103, USA
[6]Division of Digestive Diseases, Emory University School of Medicine, Atlanta, GA 30322, USA
[7]Department of Internal Medicine, University of Missouri-Kansas City, Kansas City, MO 64108, USA
[8]Division of Gastroenterology, Hepatology & Motility, The University of Kansas School of Medicine, Kansas City, KS 66103, USA

[†]Contributed equally to the manuscript

Supplemental File Index

1. Supplemental S1: Preferred Reporting Items for Systematic Reviews and Meta-analyses (PRISMA) checklist
2. Supplemental S2: Research Question, PICO, MeSH, Keywords and Search Strategy
3. Supplemental S3: GRADE assessment of included studies
4. Supplemental S4: Overview of AI platforms used in the included trials.
5. Supplemental S5: Leave one-out sensitivity testing for PDR
6. Supplemental S6: Egger's test for PDR
7. Supplemental S7: Funnel plots to check for publication bias for PDR
8. Supplemental S8: Trim-and-Fill Analysis for PDR
9. Supplemental S9: Leave one-out sensitivity testing for ADR
10. Supplemental S10: Egger's test for ADR
11. Supplemental S11: Funnel plots to check for publication bias for ADR
12. Supplemental S12: Trim-and-Fill Analysis for ADR
13. Supplemental S13: Leave one-out sensitivity testing for withdrawal time

Supplemental S1: Preferred Reporting Items for Systematic Reviews and Meta-analyses (PRISMA) checklist

PRISMA Checklist

Section and Topic	Item #	Checklist item	Location where item is reported
**TITLE**			
Title	1	Identify the report as a systematic review.	1
**ABSTRACT**			
Abstract	2	See the PRISMA 2020 for Abstracts checklist.	3
**INTRODUCTION**			
Rationale	3	Describe the rationale for the review in the context of existing knowledge.	4
Objectives	4	Provide an explicit statement of the objective(s) or question(s) the review addresses.	5
**METHODS**			
Eligibility criteria	5	Specify the inclusion and exclusion criteria for the review and how studies were grouped for the syntheses.	6
Information sources	6	Specify all databases, registers, websites, organizations, reference lists and other sources searched or consulted to identify studies. Specify the date when each source was last searched or consulted.	5
Search strategy	7	Present the full search strategies for all databases, registers and websites, including any filters and limits used.	6
Selection process	8	Specify the methods used to decide whether a study met the inclusion criteria of the review, including how many reviewers screened each record and each report retrieved, whether they worked independently, and if applicable, details of automation tools used in the process.	6
Data collection process	9	Specify the methods used to collect data from reports, including how many reviewers collected data from each report, whether they worked independently, any processes for obtaining or confirming data from study investigators, and if applicable, details of automation tools used in the process.	6
Data items	10a	List and define all outcomes for which data were sought. Specify whether all results that were compatible with each outcome domain in each study were sought (e.g. for all measures, time points, analyses), and if not, the methods used to decide which results to collect.	7
	10b	List and define all other variables for which data were sought (e.g. participant and intervention characteristics, funding sources). Describe any assumptions made about any missing or unclear information.	7

Study risk of bias assessment	11	Specify the methods used to assess risk of bias in the included studies, including details of the tool(s) used, how many reviewers assessed each study and whether they worked independently, and if applicable, details of automation tools used in the process.	7
Effect measures	12	Specify for each outcome the effect measure(s) (e.g. risk ratio, mean difference) used in the synthesis or presentation of results.	7
Synthesis methods	13a	Describe the processes used to decide which studies were eligible for each synthesis (e.g. tabulating the study intervention characteristics and comparing against the planned groups for each synthesis (item #5)).	6
	13b	Describe any methods required to prepare the data for presentation or synthesis, such as handling of missing summary statistics, or data conversions.	7
	13c	Describe any methods used to tabulate or visually display results of individual studies and syntheses.	7
	13d	Describe any methods used to synthesize results and provide a rationale for the choice(s). If meta-analysis was performed, describe the model(s), method(s) to identify the presence and extent of statistical heterogeneity, and software package(s) used.	7
	13e	Describe any methods used to explore possible causes of heterogeneity among study results (e.g. subgroup analysis, meta-regression).	7
	13f	Describe any sensitivity analyses conducted to assess robustness of the synthesized results.	7
Reporting bias assessment	14	Describe any methods used to assess risk of bias due to missing results in a synthesis (arising from reporting biases).	7
Certainty assessment	15	Describe any methods used to assess certainty (or confidence) in the body of evidence for an outcome.	7

PRISMA 2020 Checklist

Section and Topic	Item #	Checklist item	Location where item is reported
**RESULTS**			
Study selection	16a	Describe the results of the search and selection process, from the number of records identified in the search to the number of studies included in the review, ideally using a flow diagram.	6
	16b	Cite studies that might appear to meet the inclusion criteria, but which were excluded, and explain why they were excluded.	-
Study characteristics	17	Cite each included study and present its characteristics.	8

Risk of bias in studies	18	Present assessments of risk of bias for each included study.	Supplemental file
Results of individual studies	19	For all outcomes, present, for each study: (a) summary statistics for each group (where appropriate) and (b) an effect estimates and its precision (e.g. confidence/credible interval), ideally using structured tables or plots.	-
Results of syntheses	20a	For each synthesis, briefly summarize the characteristics and risk of bias among contributing studies.	-
	20b	Present results of all statistical syntheses conducted. If meta-analysis was done, present for each the summary estimate and its precision (e.g. confidence/credible interval) and measures of statistical heterogeneity. If comparing groups, describe the direction of the effect.	-
	20c	Present results of all investigations of possible causes of heterogeneity among study results.	-
	20d	Present results of all sensitivity analyses conducted to assess the robustness of the synthesized results.	-
Reporting biases	21	Present assessments of risk of bias due to missing results (arising from reporting biases) for each synthesis assessed.	-
Certainty of evidence	22	Present assessments of certainty (or confidence) in the body of evidence for each outcome assessed.	-
**DISCUSSION**			
Discussion	23a	Provide a general interpretation of the results in the context of other evidence.	14-16
	23b	Discuss any limitations of the evidence included in the review.	16
	23c	Discuss any limitations of the review processes used.	16
	23d	Discuss implications of the results for practice, policy, and future research.	17
**OTHER INFORMATION**			
Registration and protocol	24a	Provide registration information for the review, including register name and registration number, or state that the review was not registered.	-
	24b	Indicate where the review protocol can be accessed, or state that a protocol was not prepared.	-
	24c	Describe and explain any amendments to information provided at registration or in the protocol.	-
Support	25	Describe sources of financial or non-financial support for the review, and the role of the funders or sponsors in the review.	-

Competing interests	26	Declare any competing interests of review authors.	-
Availability of data, code and other materials	27	Report which of the following are publicly available and where they can be found: template data collection forms; data extracted from included studies; data used for all analyses; analytic code; any other materials used in the review.	-

From: Page MJ, McKenzie JE, Bossuyt PM, Boutron I, Hoffmann TC, Mulrow CD, et al. The PRISMA 2020 statement: an updated guideline for reporting systematic reviews. BMJ 2021;372:n71. doi: 10.1136/bmj.n71

For more information, visit: http://www.prisma-statement.org/

Supplemental S2: Research Question, PICO, MeSH, Keywords, and Search Strategy

Research Question: Does real-time AI-assisted colonoscopy improve ADR and PDR compared with standard colonoscopy in adults undergoing colorectal cancer screening?

PICO:

Population: Patients undergoing colonoscopy for colorectal cancer screening or diagnostic evaluation

Intervention: Real-time AI-assisted colonoscopy (computer-aided detection systems)

Comparison: Standard colonoscopy without AI assistance

Outcome:

1) **Primary outcome:** Polyp Detection Rate (PDR) and Adenoma Detection Rate (ADR).

2) Secondary outcomes: Withdrawal time

Study type: Randomized Controlled Trials (RCTs) comparing AI-assisted and standard colonoscopy.

MeSH Terms & Keywords:

- Colorectal Neoplasms [MeSH]
- Colonoscopy [MeSH]
- Colorectal cancer OR Colon cancer OR CRC
- Artificial Intelligence [MeSH]
- Computer-Aided Detection (CADe)
- Deep Learning OR Machine Learning
- Polyp detection rate (PDR)
- Adenoma detection rate (ADR)

Detailed search strategy for each of the included databases.

**Database**	**Search Strategy**	**Articles retrieved**
PubMed	((“Colorectal Neoplasms”[Mesh] OR “Colorectal cancer” OR “Colon cancer” OR “CRC”) AND (“Artificial Intelligence”[Mesh] OR “AI” OR “Computer-Aided Detection” OR “CADe” OR “Deep Learning” OR “Machine Learning”) AND (“Colonoscopy”[Mesh] OR	310

	“Colonoscopy” OR “Endoscopy” OR “Polyp Detection” OR “Adenoma Detection”))	
Embase	(‘colorectal cancer’/exp OR ‘colon cancer’ OR ‘CRC’) AND (‘artificial intelligence’/exp OR ‘computer aided detection’ OR ‘CADe’ OR ‘machine learning’ OR ‘deep learning’) AND (‘colonoscopy’/exp OR ‘endoscopy’ OR ‘polyp detection’ OR ‘adenoma detection’)	70
Cochrane Library	(“computer aided diagnosis” AND “colorectal cancer” AND “colonoscopy” AND “artificial intelligence” OR “colorectal polyps”)	498 (3 selected)
Google Scholar	Colorectal cancer OR colon cancer OR CRC OR artificial intelligence OR CADe OR adenoma detection rate OR polyp detection rate OR colonoscopy	21,700 (41 filtered)
ClinicalTrials.gov	Colorectal cancer AND Computer aided detection OR AI-assisted colonoscopy	59
CINAHL	Colorectal cancer AND (Computer aided detection OR Artificial Intelligence) AND Colonoscopy	81

Supplemental S3: GRADE assessment of included studies.

Certainty assessment							№ of patients		Effect		Certainty	Importance
№ of studies	Study design	Risk of bias	Inconsistency	Indirectness	Imprecision	Other considerations	AI colonoscopy	Standard colonoscopy	Relative (95% CI)	Absolute (95% CI)		
**Adenoma Detection Rate (assessed with: colonoscopy)**												
9	randomised trials	serious [a]	not serious	not serious	not serious	none	1996/4249 (47.0%)	1794/4238 (42.3%)	**RR 1.24** (0.98 to 1.58)	**102 more per 1,000** (from 8 fewer to 246 more)	⨁⨁⨁◯ Moderate[a]	IMPORTANT
**Polyp Detection Rate (assessed with: colonoscopy)**												
12	randomised trials	serious [a]	not serious	not serious	not serious	none	3161/5640 (56.0%)	2815/5627 (50.0%)	**RR 1.31** (1.08 to 1.59)	**155 more per 1,000** (from 40 more to 295 more)	⨁⨁⨁◯ Moderate[a]	IMPORTANT
**Withdrawal Time**												
11	randomised trials	serious [a]	not serious	not serious	not serious	none			**Mean difference 0.35** (-0.06 to 0.76)	**-- per 1,000** (from -- to --)	⨁⨁⨁◯ Moderate[a]	NOT IMPORTANT

CI: confidence interval; **RR:** risk ratio

Explanations

a. Risk of bias was judged as serious because of the moderate quality of the included randomized controlled trials

Supplemental S4: Overview of AI platforms used in the included trials.

Study (Author, Year)	AI Platform	Real-Time	Deployment Method	Training Dataset	Validation Approach	Regulatory Status	AI Method
Wang et al., 2020 [9]	Custom CADe	Yes	Bounding box	Institutional dataset	Internal validation	Research use	Deep learning (CNN)
Xu et al., 2021 [20]	Custom CADe	Yes	Bounding box	Annotated videos	Dual review	Research use	Custom CADe (RestinaNet-based)
Yao et al., 2022 [21]	EndoAngel	Yes	Bounding box + quality indicators	Large-scale Chinese dataset	Internal and external validation	Approved (China)	EndoAngel (CADe + CAQ)
Shaukat et al., 2022 [5]	SKOUT™	Yes	Bounding box	US multicenter dataset	Prospective validation	FDA-cleared	Proprietary (Iterative Scopes)
Brown et al., 2022 [14]	EndoScreener	Yes	Bounding box	Chinese + US video training	External testing	FDA-cleared	Deep learning (CNN)
Gong et al., 2020 [11]	Custom CADe	Yes	Bounding box	Video training set	Blinded validation	Research use	Deep neural networks + perceptual hash

Liu et al., 2019 [16]	In-house CADe	Yes	Visual alert	Endoscopy video frames	Real-time performance	Institutional	Deep learning (custom CNN)
Luo et al., 2021 [17]	CADe tool	Yes	Boundin g box	Internal dataset	Retrospective review	Research use	Deep learning (custom CNN)
Mangas et al., 2023 [18]	CADILLA C CADe	Yes	Box + sound alert	Spanish consortium	Multicenter pilot	CE-certified	Deep learning (Spanish consortium)
Wallace et al., 2022 [7]	SKOUT-like	Yes	Boundin g box	US/Europe centers	Blinded assessment	FDA/CE-pending	Deep learning (Cosmo AI, proprietary)
Shen et al., 2021 [19]	Real-time CADe	Yes	Inline video overlay	Endoscopy frames	Dual-rater analysis	Research use	YOLO v3 CNN
Yamaguchi et al., 2024 [15]	In-house CADe	Yes	Boundin g box	Hospital dataset	Blinded review	Institutional	Deep learning (Fujifilm proprietary)

Supplemental S5: Leave-one-out sensitivity analysis for PDR. Each point represents the pooled log OR and 95% CI when one study is omitted. [5,7,9,11,14–21].

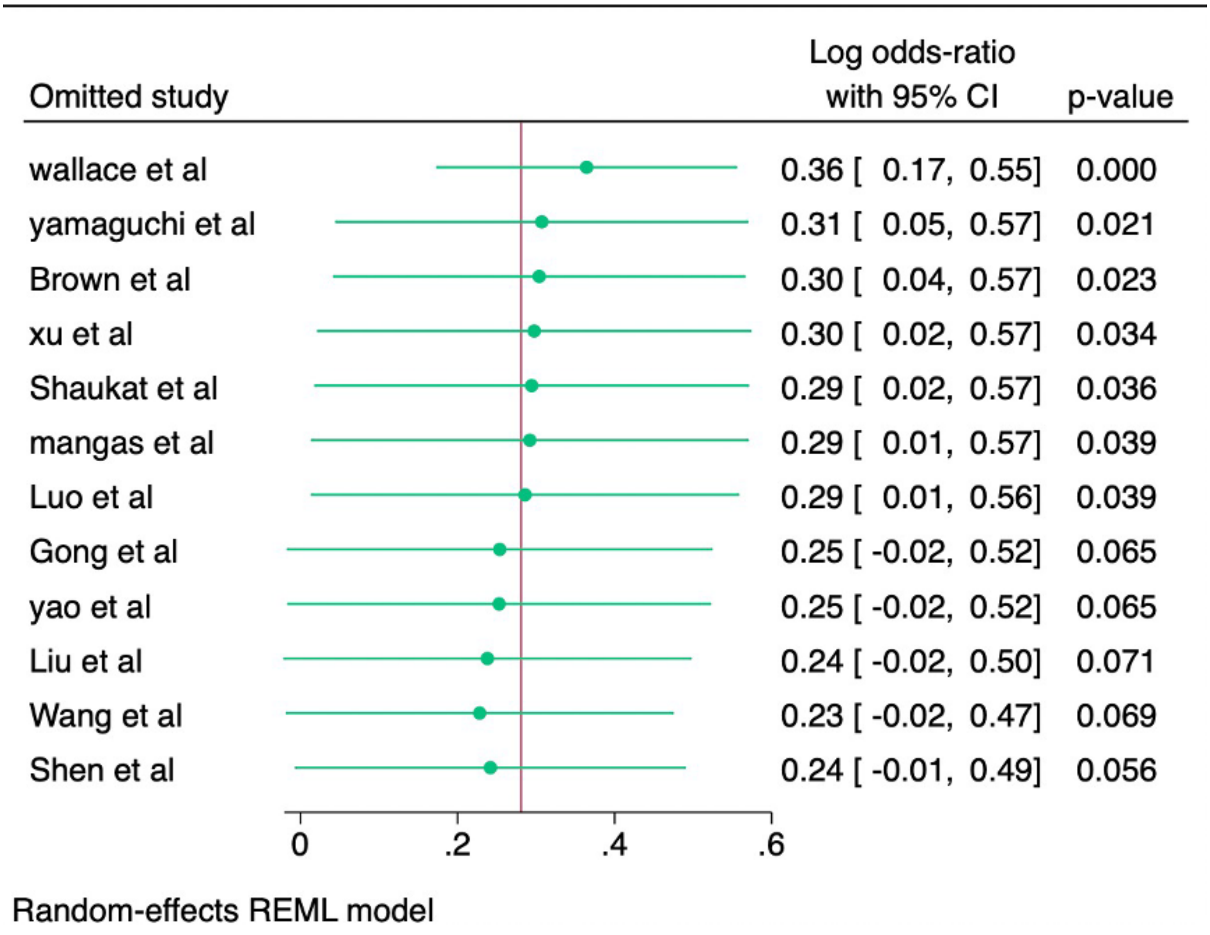

Supplemental S6: Egger's regression test for small-study effects in the meta-analysis of PDR.

```
Egger's test for small-study effects:
Regress standard normal deviate of intervention
  effect estimate against its standard error

.
Number of studies =  12                              Root MSE      =   2.482
------------------------------------------------------------------------------
     Std_Eff | Coefficient  Std. err.      t    P>|t|     [95% conf. interval]
-------------+----------------------------------------------------------------
       slope |   .2526165   .2231268     1.13   0.284    -.2445409    .7497739
        bias |   .2438079   1.617853     0.15   0.883    -3.360992    3.848608
------------------------------------------------------------------------------

Test of H0: no small-study effects          P = 0.883
```

Supplemental S7: Funnel plot assessing publication bias in studies reporting PDR. The distribution of effect sizes is symmetric, indicating low risk of bias.

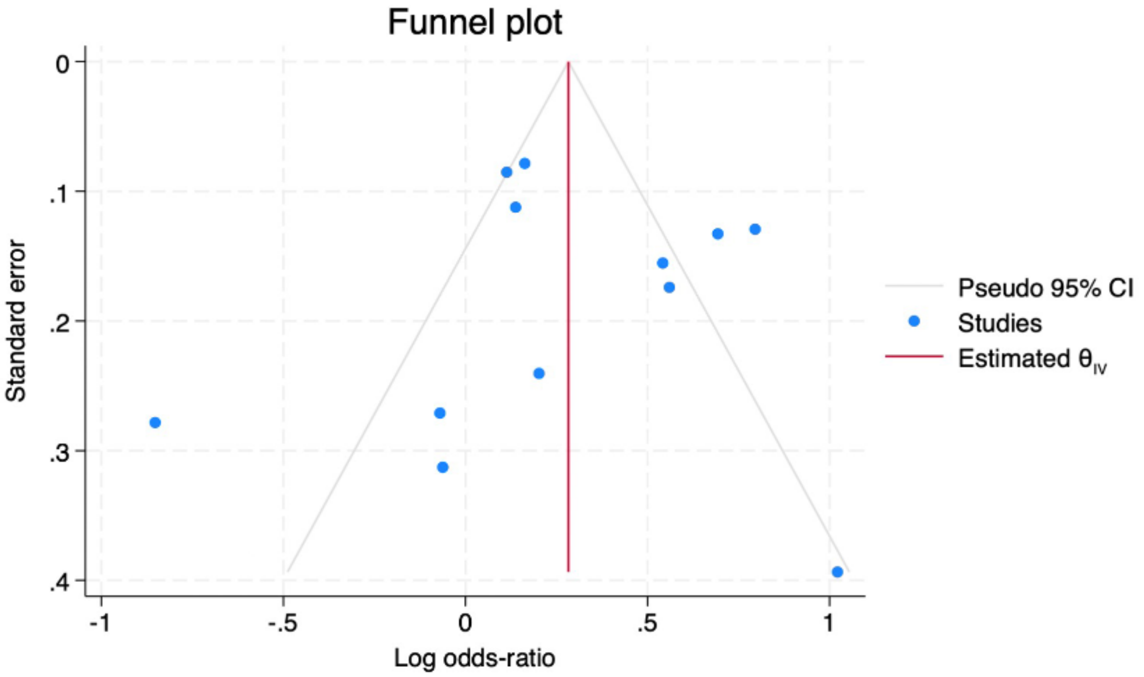

Supplemental S8: Trim-and-Fill Plot for PDR

```
Nonparametric trim-and-fill analysis of publication bias
Linear estimator, imputing on the left

Iteration                             Number of studies =     12
  Model: Random-effects                        observed =     12
 Method: REML                                  imputed =       0

Pooling
  Model: Random-effects
 Method: REML

---------------------------------------------------------------
              Studies |     Effect size    [95% conf. interval]
----------------------+----------------------------------------
             Observed |           0.265       0.034       0.497
   Observed + Imputed |           0.265       0.034       0.497
---------------------------------------------------------------
```

Supplemental S9 :Leave-one-out sensitivity analysis for ADR. [5,7,9,11,14–16,18,21]

Omitted study	Effect-size	[95% conf.	interval]	p-value
yao et al	0.204	0.111	0.298	0.000
mangas et al	0.292	0.172	0.411	0.000
yamaguchi et al	0.225	0.132	0.318	0.000
wallace et al	0.248	0.155	0.341	0.000
Gong et al	0.189	0.096	0.283	0.000
Wang et al	0.206	0.109	0.303	0.000
Liu et al	0.147	0.050	0.245	0.003
Brown et al	0.217	0.124	0.310	0.000
Shaukat et al	0.227	0.126	0.329	0.000
theta	0.215	0.123	0.306	0.000

Supplemental S10: Egger’s regression test for small-study effects in the ADR meta-analysis.

```
Egger's test for small-study effects:
Regress standard normal deviate of intervention
  effect estimate against its standard error

.
Number of studies =   9                             Root MSE      =    2.426
------------------------------------------------------------------------------
     Std_Eff | Coefficient  Std. err.      t    P>|t|     [95% conf. interval]
-------------+----------------------------------------------------------------
       slope |   .2009778     .24998     0.80   0.448    -.3901309    .7920865
        bias |   .1011559   1.770254     0.06   0.956     -4.08483    4.287142
------------------------------------------------------------------------------

Test of H0: no small-study effects          P = 0.956
```

Supplemental S11: Funnel plot assessing publication bias in ADR.

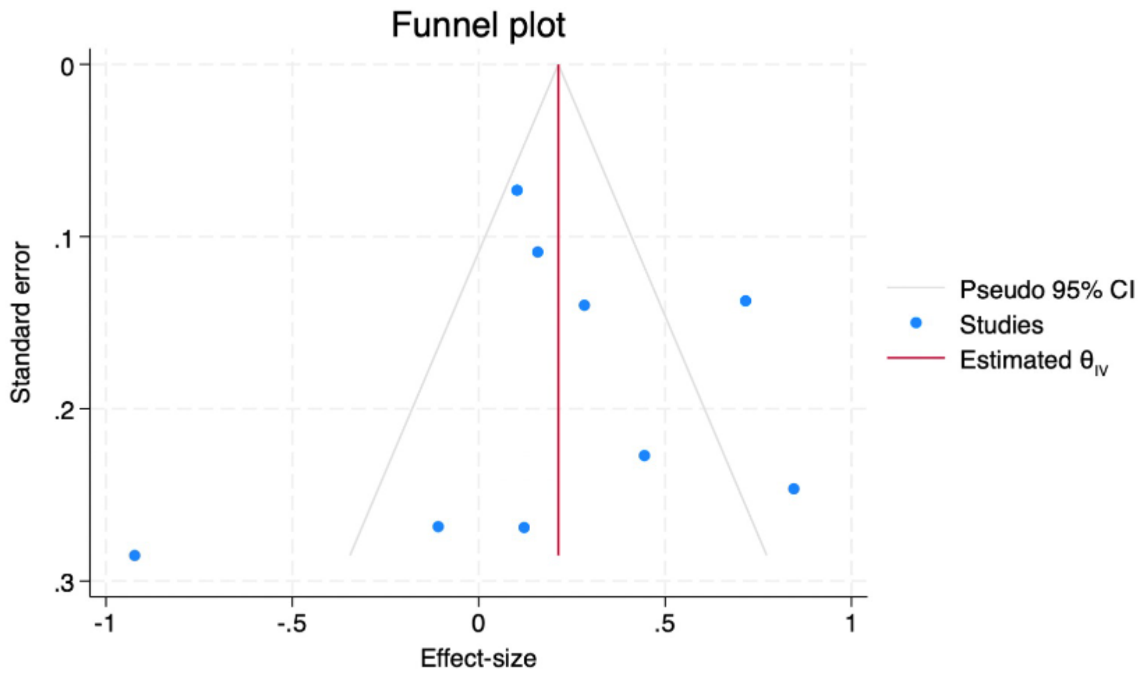

Supplemental S12: Trim-and-Fill Analysis for ADR

```
Nonparametric trim-and-fill analysis of publication bias
Linear estimator, imputing on the left

Iteration                            Number of studies =      9
  Model: Random-effects                       observed =      9
 Method: REML                                 imputed =      0

Pooling
  Model: Random-effects
 Method: REML

---------------------------------------------------------------
              Studies |    Effect size    [95% conf. interval]
----------------------+----------------------------------------
             Observed |          0.205      -0.098       0.508
  Observed + Imputed |          0.205      -0.098       0.508
---------------------------------------------------------------
```

Supplemental S13: Leave-one-out sensitivity analysis evaluating the impact of individual studies on the pooled mean difference in withdrawal time between AI-assisted and standard colonoscopy. [5,7,9,11,15–21].

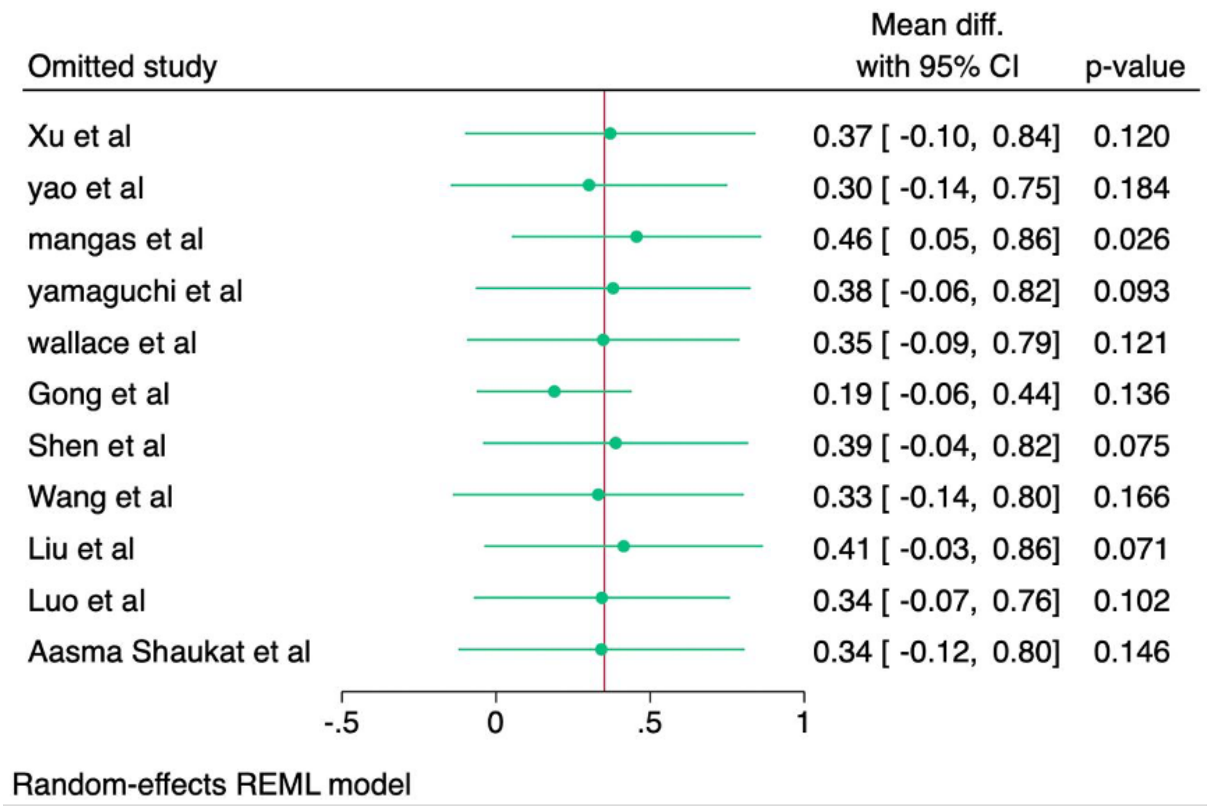

Supplement: Supplementary file 1 [file healthcare-13-02517-s001.zip › healthcare-3803757 Supplemental File-Revised.pdf]
